# Botulinum toxin type A promotes microglial M2 polarization and suppresses chronic constriction injury-induced neuropathic pain through the P2X7 receptor

**DOI:** 10.1186/s13578-020-00405-3

**Published:** 2020-03-23

**Authors:** Xianwei Gui, Hansen Wang, Lanxiang Wu, Sheng Tian, Xuan Wang, Heqing Zheng, Wei Wu

**Affiliations:** Department of Neurology, The Second Affiliated Hospital of Nanchang University, No. 1 Minde Road, Nanchang, 330006 Jiangxi China

**Keywords:** Botulinum toxin type A, Neuropathic pain, Microglia, M1/M2 polarization, P2X7 receptor

## Abstract

**Background:**

Switching microglial polarization from the pro-inflammatory M1 phenotype to the anti-inflammatory M2 phenotype represents a novel therapeutic strategy for neuropathic pain (NP). This study aims to investigate whether botulinum toxin type A (BTX-A) regulates microglial M1/M2 polarization by inhibiting P2X7 expression in a rat model of NP.

**Results:**

The BTX-A administration elevated pain threshold, induced microglial polarization toward the M2 phenotype, and decreased P2X7 protein level in a rat model of NP induced by chronic compression injury (CCI). Lipopolysaccharide (LPS) was used to activate HAPI rat microglial cells as an in vitro inflammatory model and we demonstrated that BTX-A promoted microglial M2 polarization in LPS-stimulated HAPI microglial cells through suppressing P2X7.

**Conclusions:**

Our results indicate that BTX-A promotes microglial M2 polarization and suppresses CCI-induced NP through inhibiting P2X7 receptor. These findings provide new insights into the mechanism of BTX-A in relieving NP.

## Background

Neuropathic pain (NP), characterized by allodynia, hyperalgesia and spontaneous pain, is a chronic condition that can develop after a lesion or disease affecting the somatosensory system [1, 2]. NP seriously affects the health and life quality of patients. So far, there is still a lack of effective treatment for NP [3, 4]. Microglia, the resident macrophage cells of the central nervous system, has been shown to mediate neuroinflammation [5]. Microglia activation and subsequent pro-inflammatory responses play an important role in the development of NP [6].

Microglia can be activated in a polarizing manner into a classical phenotype (pro-inflammatory, M1) or an alternative phenotype (anti-inflammatory, M2). The M1 phenotype is characterized by increased expression of several proteins including inducible NO synthase (iNOS) and CD68, as well as increased secretion of several pro-inflammatory factors such as interleukin (IL)-1β, IL-6, and tumor necrosis factor (TNF)-α [7, 8]. The M2 phenotype is characterized by increased expression of several proteins such as arginase-1 (Arg-1) and mannose receptor (MR/CD206), as well as increased production of anti-inflammatory cytokines such as IL-4 and IL-10 [5, 9]. The microglial polarization toward the pro-inflammatory M1 phenotype often occurs during NP. Convincing evidence has indicated that modulation of inflammation by inhibition of M1 polarization can be a strategy for treatment of NP [10–13].

Botulinum toxin type A (BTX-A) is an exotoxin released by Gram-positive anaerobic *Clostridium botulinum* and has been widely used in the treatment of dystonia and in aesthetic field [14, 15]. Clinical studies have confirmed that BTX-A can effectively relieve NP with mild adverse reactions [16, 17]. However, the exact mechanism of BTX-A action in NP is still undefined. Increasing studies have suggested that the analgesic effect of BTX-A is mediated through neurons and glial cells, especially microglia [18]. However, no literature has reported the effect of BTX-A on microglial polarization.

P2X7 receptor (also called P2X7) is a member of the purinergic ion channel receptor (P2X) family (P2X1–P2X7), which is predominantly present on microglia, astrocytes and neurons. P2X7 has been suggested to be involved in the pain transmission and the occurrence of NP [19]. Studies have reported that BTX-A can inhibit the expression of P2X3 receptor in dorsal root ganglion (DRG) in rats with NP [20], but the effect of BTX-A on P2X7 receptor has not been reported. Recently, P2X7 receptor has been proposed as a marker of M1 microglia [21]. The involvement of P2X7 receptor in microglial M1 polarization is further supported by in vitro results that microglial M1 polarization could be avoided by inhibition of P2X7 receptor in ischaemic conditions [22]. Thus, in the present study, we attempt to investigate whether microglial polarization plays a role in the analgesic effect of BTX-A. We also explored whether BTX-A regulates microglial polarization via P2X7 receptor.

## Materials and methods

### Animals

Specific pathogen-free (SPF) Sprague–Dawley male rats (weight 200–250 g) were used in our study. The animals were kept at controlled temperature (22 ± 2 °C) with a 12 h light–dark cycle. All animal procedures were in compliance with the National Institutes of Health Guidelines for the Care and Use of Laboratory Animals. This study was approved by the Ethics Committee of the Second Affiliated Hospital of Nanchang University.

### Establishment of a rat model of NP

A rat model of NP was induced by chronic compression injury (CCI). Briefly, each rat was anesthetized by intraperitoneal injection of 2% pentobarbital sodium (50 mg/kg) before surgical procedures. After skin preparation, approximately 1-cm incision was made along the middle of the lower margin of the right femur. The sciatic nerve was exposed by blunt separation of muscles. Then 4 loose knots at 1 mm-intervals were made with 4–0 chromium catgut. The epineurium was slightly compressed, and the tightness was to the slight twitching of the toes. Subsequently, the muscle fascia and skin were routinely sutured. If the rats after CCI showed limping, the right hindlimb showed slight valgus, sometimes licking, hanging and other hindlimb protection behaviors, it indicated that the NP model was successfully established. In Sham-operated group, the sciatic nerve was exposed for 2–3 min without knotting, and then the muscle fascia and skin were sutured.

### Animal groups

Sprague–Dawley rats were randomly divided into the following four groups (n = 10/group): Sham, NP, BTX-A-10, and BTX-A-20 group. Rats in the BTX-A-10 and BTX-A-20 groups were given a subcutaneous injection of 10 U/kg BTX-A (Allergan Pharmaceuticals Ireland) and 20 U/kg BTX-A respectively into the metatarsal surface 3 d following model establishment of CCI. Rats in the Sham and NP were injected with normal saline instead of BTX-A.

### Pain threshold determination

Measurement of mechanical withdrawal threshold (MWT) and thermal withdrawal latency (TWL) was performed at 0, 3, 5, 7, 10, 12, 14 days after induction of CCI. The MWT value of the injured side of rats was measured by using an electronic von Frey device (series 2390; IITC-Life Science Instruments, Woodland Hills, CA, USA). The plantar of the right hind limb of the quiet rats was gradually pressurized until a withdrawal response of evasive leg lifting occurred. The maximum pressure was recorded and expressed in grams. The experiment was repeated for 6 times at an interval of 5 min. The MWT was calculated as the mean of 4 values after removing the maximum and minimum values.

The TWL value of the injured side of rats was measured using an automatic Plantar Test (No. 37370; Ugo Basile, Varese, Italy). The instrument emitted infrared light to irradiate the plantar of the right hind limb of rats. The instrument automatically cut off the supply of heat and recorded the time from the beginning of irradiation to the occurrence of foot shrinkage escape. The experiment was repeated for 5 times at an interval of 10 min. The TWL was calculated as the mean of 3 values after removing the maximum and minimum values.

### Tissue samples

Fourteen days after CCI modeling, the rats were sacrificed under anesthesia with pentobarbital sodium (50 mg/kg). The limbs were fixed, the thoracic cavity was fully exposed, and the aorta was intubated through the left ventricle. After the liver was cut off, the heart was perfused with 0.9% NaCl solution (200 mL), followed by infusion with 4% paraformaldehyde in PBS buffer (200 mL). The L4–L6 segments of the spinal cord of rats were isolated and fixed in 4% paraformaldehyde, dehydrated in gradient ethanol, and embedded in paraffin for immunofluorescent detection. The remaining L4–L6 spinal cord segments were stored at − 80 °C and used for western blot analysis.

### Immunofluorescent staining

The sections were dewaxed, rehydrated, and then immersed into citrate buffer for antigen retrieval. After that, the sections were blocked with 2% bovine serum albumin and then incubated overnight at 4 °C with the following primary antibodies: anti-ionized calcium binding adaptor molecule-1 (Iba-1) (1:100; Abcam, Cambridge, MA, USA), anti-CD68 (1:200; Santa Cruz Biotechnology, Dallas, TX, USA), anti-CD206 (1:200; Santa Cruz Biotechnology), anti-P2X7 (1:200; Invitrogen, Thermo Fisher Scientific, Inc., Waltham, MA, USA), anti- microtubule-associated protein 2 (MAP2; 1:200; Abcam), anti-CD11b (1:200; Abcam), followed by incubation with the secondary antibodies Alex Fluor® 488-labeled secondary antibody (green; 1:1000; Abcam), Alex Fluor® 647-labeled secondary antibody (red; 1:1000; Abcam) and DAPI (blue; 1:1000; Santa Cruz Biotechnology) for 1 h at room temperature. Slides were observed by a fluorescent microscope equipped with a Canon EOS digital camera.

### Flow cytometry

The L4–L6 spinal cord segments were collected and made into single cell suspension. The mononuclear cells were isolated by density gradient centrifugation using Percoll and stained with anti-CD11b. The cells were then stained with the following fluorochrome-labeled antibodies: anti-CD68-PE (Invitrogen) and anti-CD206-Alexa Fluor 647 (Abcam). The cells were then fixed and analyzed with an Accuri C6 flow cytometer (Becton Dickinson, Franklin Lakes, NJ, USA).

### Cell culture and treatment

The rat microglial cell line (HAPI) was obtained from BeNa Culture Collection (China). Cells were maintained in high glucose Dulbecco's modified Eagle's medium (Gibco, Thermo Fisher Scientific, Inc.) containing 10% fetal bovine serum (Gibco) in a humidified atmosphere of 95% air and 5% CO_2_. The cells were treated with different concentrations of BTX-A (0.01, 0.1, 1, 5, 10, 50, 100 nM), and the cell viability was detected by Cell Counting Kit‐8 (CCK‐8; Beyotime, Shanghai, China). The OD450 values were determined by an enzyme-labeled analyzer. Lipopolysaccharide (LPS; 100 ng/mL; Sigma-Aldrich, St. Louis, MO, USA) was used to activate HAPI microglial cells.

### Cell transfection

The full-length sequences of P2X7 were subcloned into pcDNA3.1 vector (Invitrogen) referred as pcDNA3.1-P2X7, whereas empty pcDNA3.1 plasmid acted as negative control. Cells were transfected with these vectors using Lipofectamine 2000™ (Invitrogen). At 48 h post-transfection, HAPI cells were harvested to examine the overexpression efficiency.

### Western blot

Total protein from L4–L6 spinal cord segments and cells was extracted using lysis buffer (Beyotime, Shanghai, China). The protein concentrations were determined by BCA assay. Then equal protein from cell lysates was separated by 10% SDS-PAGE gels and electro-transferred onto PVDF membranes (Millipore Corp., Billerica, MA, USA). After blocked with 5% skim milk at room temperature for 2 h, the membranes were incubated with the following primary antibodies against P2X7 (1:1000; Invitrogen) and β-actin (1:1000; Santa Cruz Biotechnology) overnight at 4 °C. The protein was detected with an enhanced chemiluminescence kit (Applygen Technologies Inc., Beijing, China). The band intensity was analyzed by Image-Pro Plus 6.0 software.

### Quantitative real-time PCR (qRT-PCR)

Total RNA was extracted from L4–L6 spinal cord segments and HAPI cells using TRIzol reagent (Invitrogen), and reverse-transcribed to cDNA using a PrimeScript RT Reagent Kit (TaKaRa, Dalian, China). The mRNA levels of iNOS and Arg-1 were examined using SYBR Premix Dimmer Eraser kit (TaKaRa) by the ABI7900 system (Applied Biosystem, Foster City, CA, USA). The relative quantification was calculated using the 2^−ΔΔct^ method. GAPDH was used as the internal control. Primers were synthesized by Sangon Biotechnology (Shanghai, China).

### Enzyme-linked immunosorbent assay (ELISA)

The levels of various cytokines including IL-6, IL-10, and TNF-α in cell supernatant were measured with their commercial ELISA kits (R&D Systems, Minneapolis, MN, USA) according to the manufacturer’s instructions.

### Statistical analysis

The data are presented as the mean ± standard deviation (SD) from three independent experiments. One-way analysis of variance was used to analyze differences among multiple groups. Statistical analyses were performed using SPSS version 20.0 (IBM, Chicago, IL, USA). P-value < 0.05 was considered significant in all the tests.

## Results

### BTX-A elevated pain threshold and promoted microglial polarization toward the M2 phenotype in NP rats

**Fig. 1:**
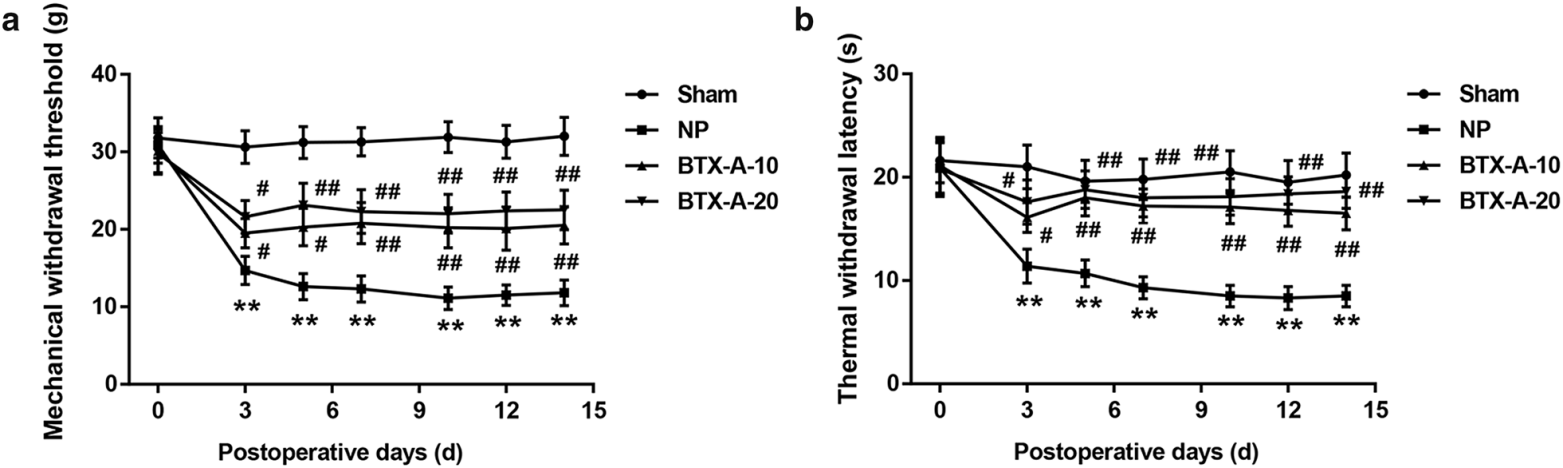
BTX-A elevated pain threshold in NP rats. Sprague–Dawley rats were randomly divided into the four groups (n = 10/group): Sham, CCI-induced NP, BTX-A-10 (rats were administered 10 U/kg BTX-A following CCI), and BTX-A-20 group (rats were administered 20 U/kg BTX-A following CCI). The mechanical withdrawal threshold (MWT; **a**) and thermal withdrawal latency (TWL; **b**) was measured at 0, 3, 5, 7, 10, 12, 14 days after induction of CCI. **P < 0.01, vs*.* Sham; ^#^P < 0.05, ^##^P < 0.01, vs*.* NP

**Fig. 2:**
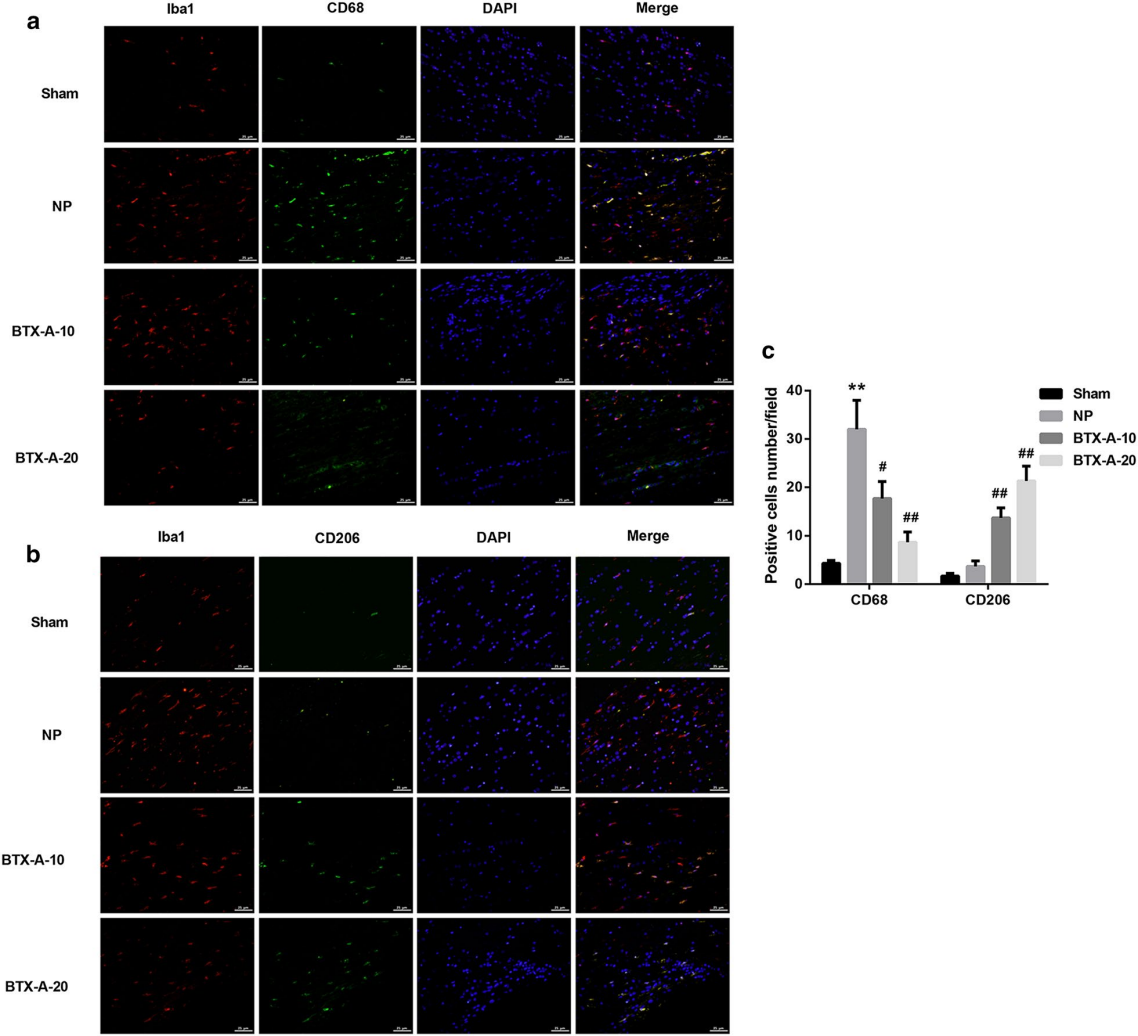
BTX-A promoted microglial activation and M2 polarization in NP rats. Sprague–Dawley rats were randomly divided into the four groups (n = 10/group): Sham, CCI-induced NP, BTX-A-10 (rats were administered 10 U/kg BTX-A following CCI), and BTX-A-20 group (rats were administered 20 U/kg BTX-A following CCI). **a**, **b** Representative immunofluorescence images in rat L4–L6 spinal cord segments fourteen days after CCI induction. DAPI (nuclei, blue), total microglia (Iba-1; red), M1-microglia phenotype (CD68; green), and M2-microglia phenotype (CD206, green). **c** Quantification of CD68-positive and CD206 positive cells per field. **P < 0.01, vs. Sham; ^#^P < 0.05, ^##^P < 0.01, vs*.* NP

**Fig. 3:**
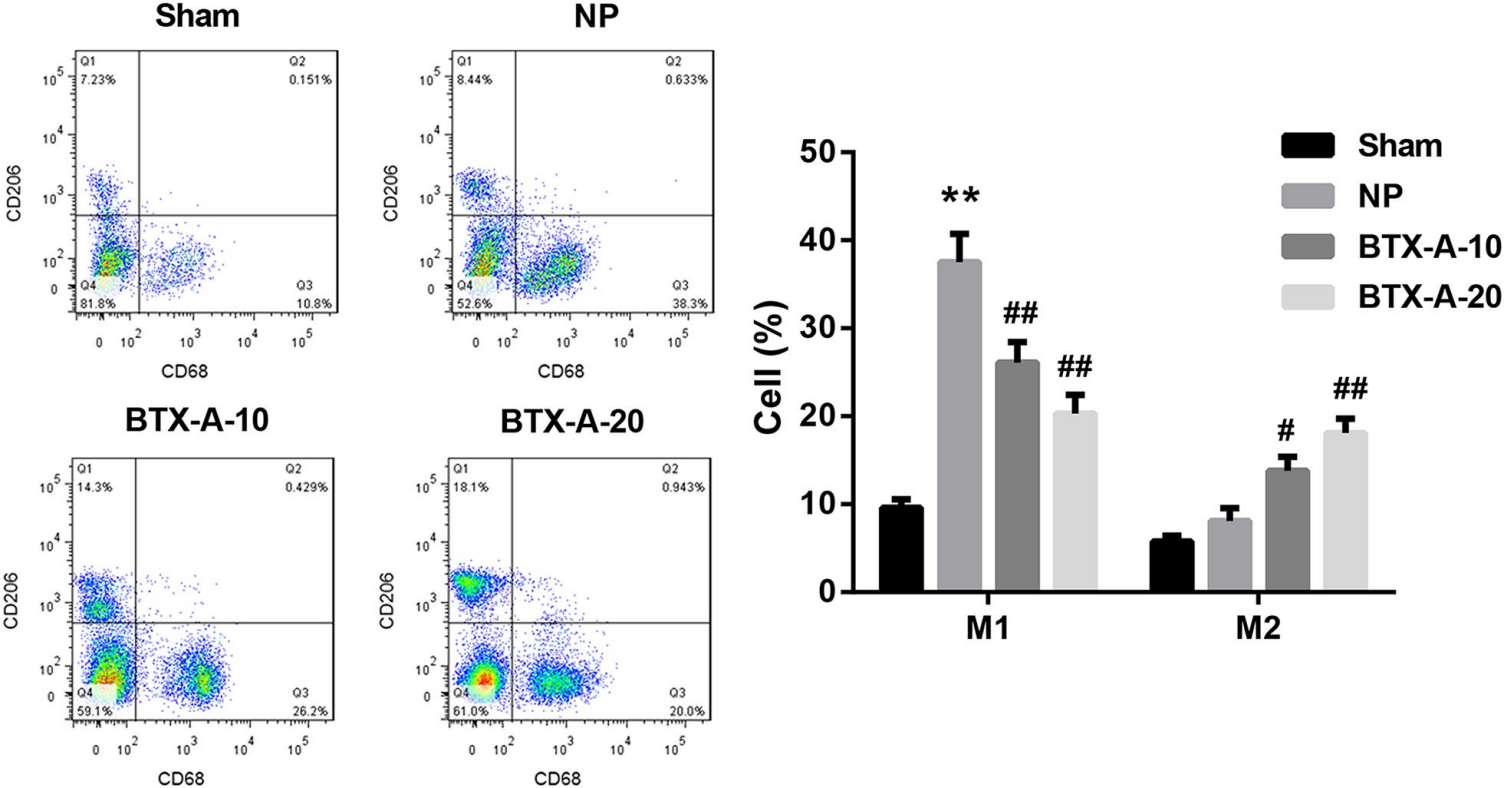
Effect of BTX-A on M1 and M2 microglia in NP rats. The proportions of both M1 and M2 microglia in L4–L6 spinal cord segments were examined by flow cytometry. **P < 0.01, vs*.* Sham; ^#^P < 0.05, ^##^P < 0.01, vs*.* NP

Compared with the sham-operated group, the MWT and TWL values were significantly decreased in the NP group. However, MWT and TWL values were notably elevated in both BTX-A-10 group and BTX-A-20 group when compared with the NP group, indicating that BTX-A administration elevated rat pain threshold post-CCI (Fig. 1a, b**)**. To explore whether BTX-A administration alters microglia polarization, we used antibodies specific to Iba-1 (a marker of microglial activation) and CD68 (a M1 marker of microglia) to identify M1, and antibodies specific to Iba-1 and CD206 (a M2 marker of microglia) to identify M2. As shown in Fig. 2a–c, a significant increase in the number of Iba-1^+^CD68^+^ cells (M1) and a slight increase in the number of Iba-1^+^CD206^+^ cells (M2) were observed in the NP group when compared with the Sham group, indicating microglial activation in the rat model of NP. Furthermore, BTX-A administration resulted in a considerable increase in the number of Iba-1^+^CD206^+^ cells but a notable decrease in that of Iba-1^+^CD68^+^ cells. Moreover, data from flow cytometry analysis further confirmed that BTX-A treatment promoted microglial polarization toward the M2 phenotype in NP rats (Fig. 3).

### BTX-A promoted microglial M2 polarization in LPS-stimulated HAPI microglial cells

**Fig. 4:**
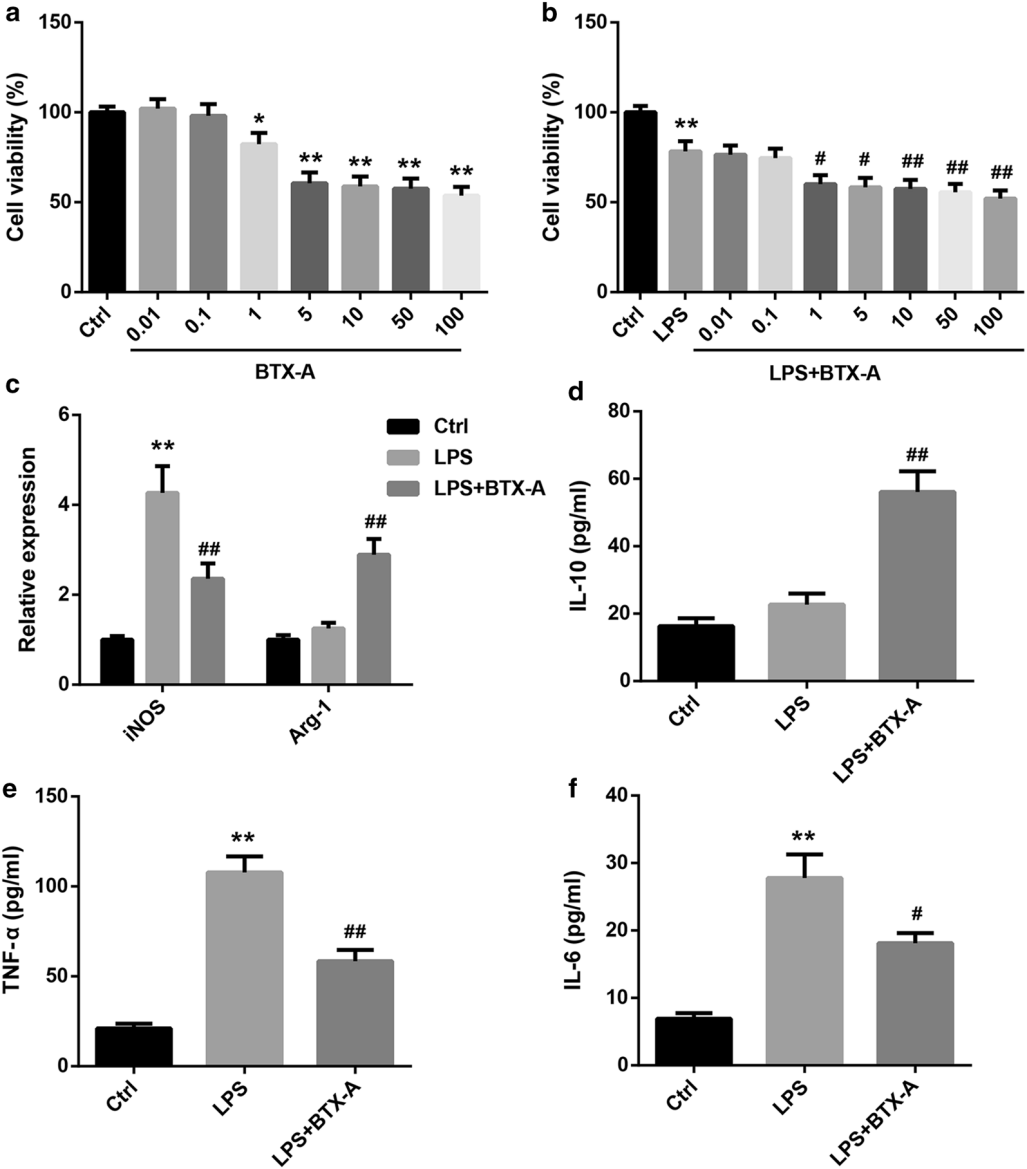
BTX-A promoted microglial M2 polarization in LPS-stimulated HAPI microglial cells. **a** Cell viability of HAPI cells treated without or with BTX-A (0.01, 0.1, 1, 5, 10, 50, 100 nM). **b** Cell viability of HAPI cells treated without or with BTX-A (0.01, 0.1, 1, 5, 10, 50, 100 nM) and LPS (100 ng/mL). Relative mRNA levels of iNOS and Arg-1 determined by qRT-PCR (**c**) and levels of M2-related IL-10 (**d**), M1-related TNF-α (**e**) and IL-6 (**f**) in HAPI cells treated without or with BTX-A (0.1 nM) and LPS (100 ng/mL). *P < 0.05, *P < 0.01, vs. Ctrl; ^#^P < 0.05, ^##^P < 0.01, vs. LPS

To corroborate the histological findings, HAPI cells were treated with BTX-A at different concentrations in the absence and presence of LPS, a known microglial activator. CCK-8 assay showed that both with or without LPS, BTX-A had no significant effect on cell viability at doses of 0.01 nM and 0.1 nM, whereas caused a significant decrease in cell viability at doses not less than 1 nM (Fig. 4a, b). Thus, 0.1 nM BTX-A was selected four subsequent experiments. We further verified whether BTX-A regulates microglial polarization. LPS treatment resulted in a marked increase in expression of iNOS (a M1 marker of microglia) and M1-related cytokines (TNF-α and IL-6). Importantly, the effect of LPS was effectively attenuated by BTX-A, indicating BTX-A inhibited the LPS-induced microglial M1 polarization (Figs. 4c, e, f). Furthermore, expression of Arg-1 (a M2 marker of microglia) and M2-associated IL-10 were greatly upregulated by BTX-A treatment, further suggesting that BTX-A promoted microglial polarization to the M2 phenotype (Fig. 4c, d).

### BTX-A decreased P2X7 expression

**Fig. 5:**
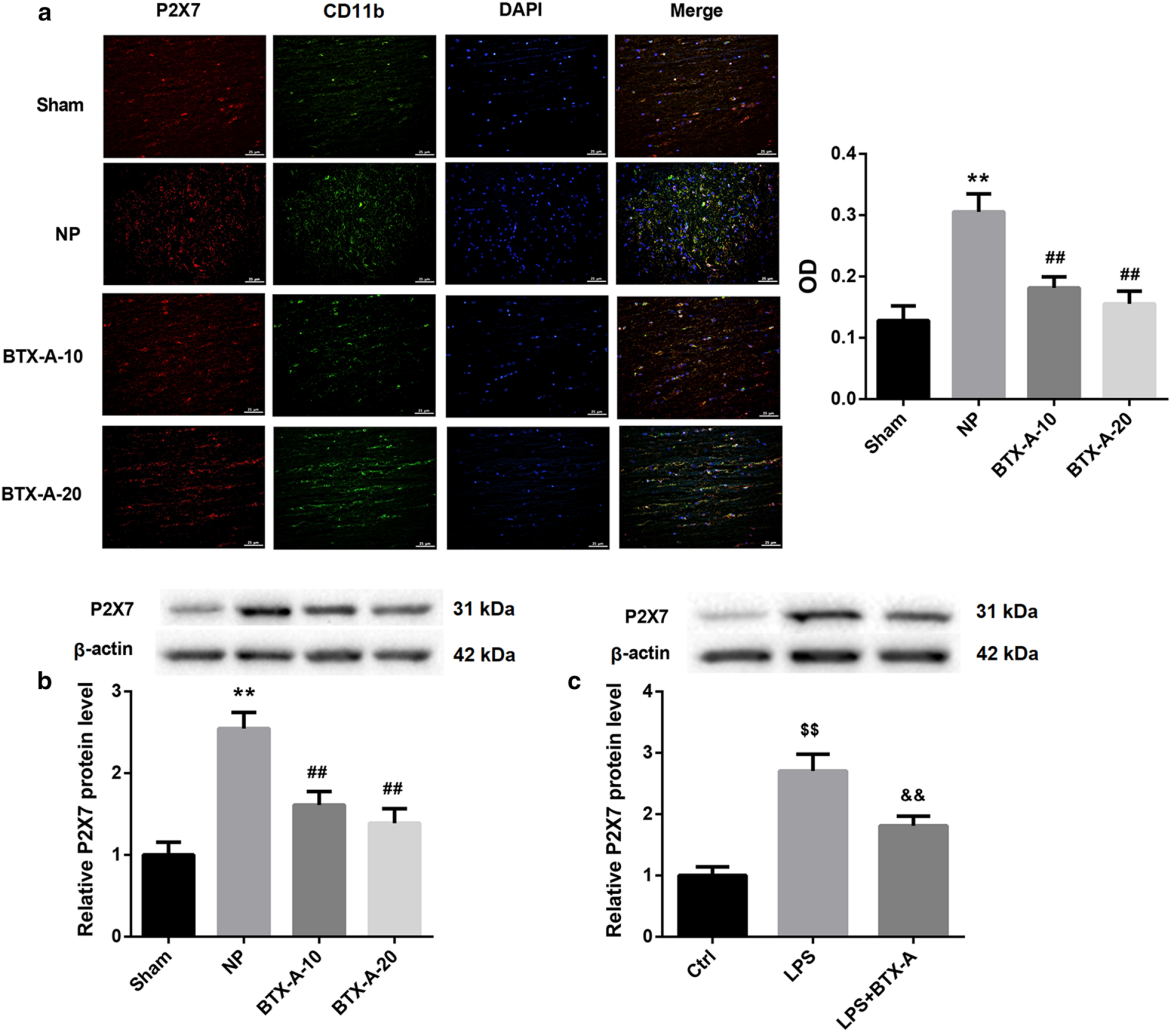
BTX-A downregulated P2X7 expression. **a** Immunofluorescence double-labeling of P2X7 and CD11b and **b** Western blot analysis of P2X7 protein level in rat L4–L6 spinal cord segments fourteen days after CCI induction in the groups of Sham, NP, BTX-A-10, and BTX-A-20. **c** Western blot analysis of P2X7 protein level in HAPI cells treated without or with BTX-A (0.1 nM) and LPS (100 ng/mL). ^**^P < 0.01, vs. Sham; ^##^P < 0.01, vs*.* NP; ^$$^P < 0.01, vs. Ctrl; ^&&^P < 0.01, vs. LPS

P2X7 immunofluorescence was not double-labeled with MAP2 (a marker for neuron). Instead, almost all P2X7-positive cells were double-labeled with CD11b (a marker for microglia), which indicated that the P2X7 expression was highly restricted to microglia but not neurons in rat L4–L6 spinal cord segments after nerve injury (Additional file 1: Fig. S1). Importantly, BTX-A administration at doses of both 10 U/kg and 20 U/kg resulted in a notable decrease in P2X7 level in rat L4–L6 spinal cord segments fourteen days after CCI induction (Fig. 5a). Western blot analysis consolidated the results from immunofluorescence (Fig. 5b). Furthermore, western blot analysis showed that BTX-A treatment also significantly reduced the LPS-induced P2X7 protein level in HAPI cells (Fig. 5c).

### BTX-A promoted microglial M2 polarization through suppressing P2X7

**Fig. 6:**
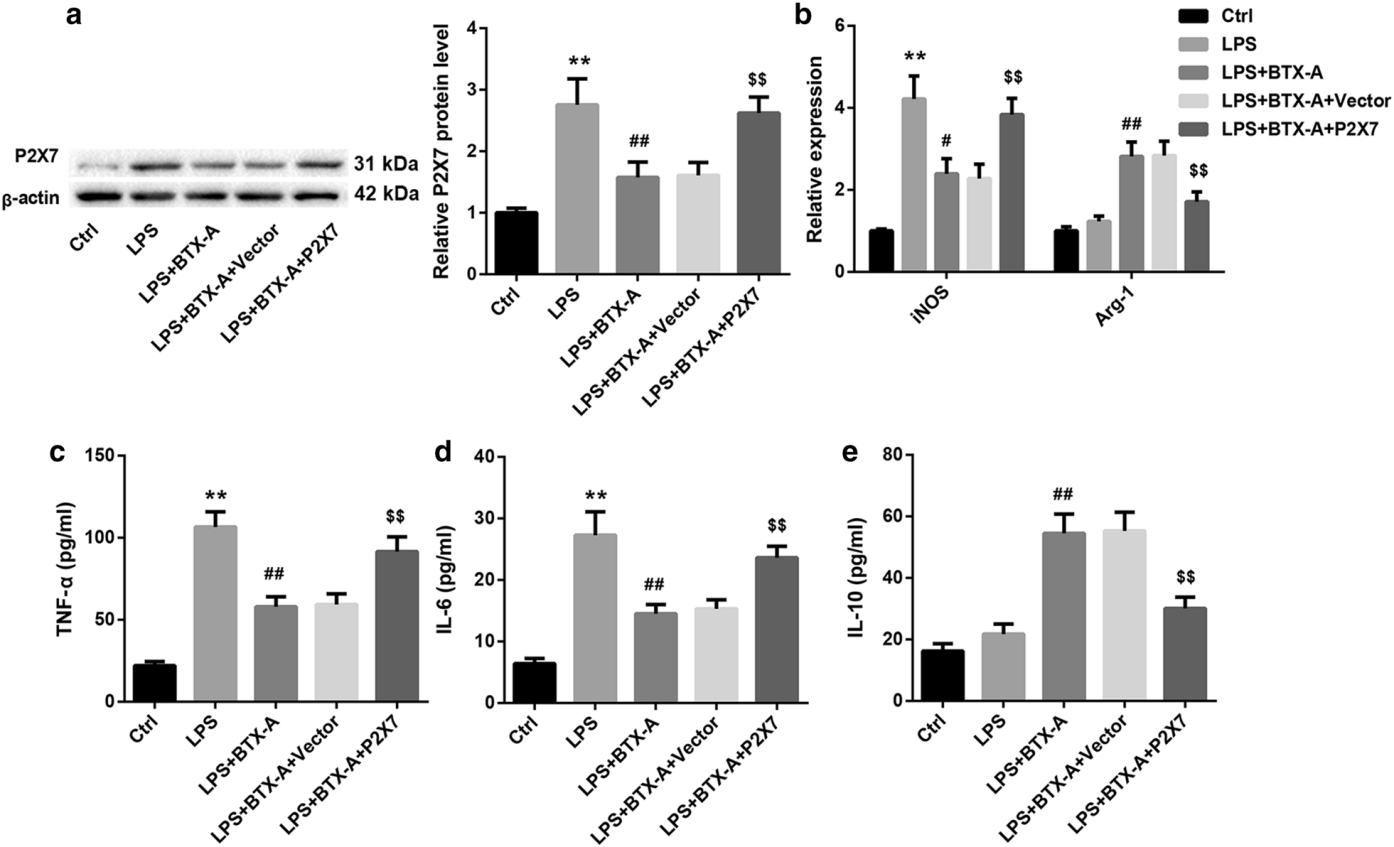
P2X7 overexpression attenuated the BTX-A-mediated promotion of microglial M2 polarization. **a** Western blot analysis of P2X7 protein level, **b** relative mRNA levels of iNOS and Arg-1 determined by qRT-PCR, and levels of M1-related TNF-α (**c**) and IL-6 (**d**) and M2-related IL-10 (**e**) in HAPI cells in the groups of Ctrl, LPS, LPS + BTX-A, LPS + BTX-A + Vector, and LPS + BTX-A + P2X7. BTX-A: 0.1 nM; LPS (100 ng/mL) was used as a microglial activator. **P < 0.01, vs. Ctrl; ^#^P < 0.05, ^##^P < 0.01, vs. LPS; ^$$^P < 0.01, vs. LPS + BTX-A + Vector

**Fig. 7:**
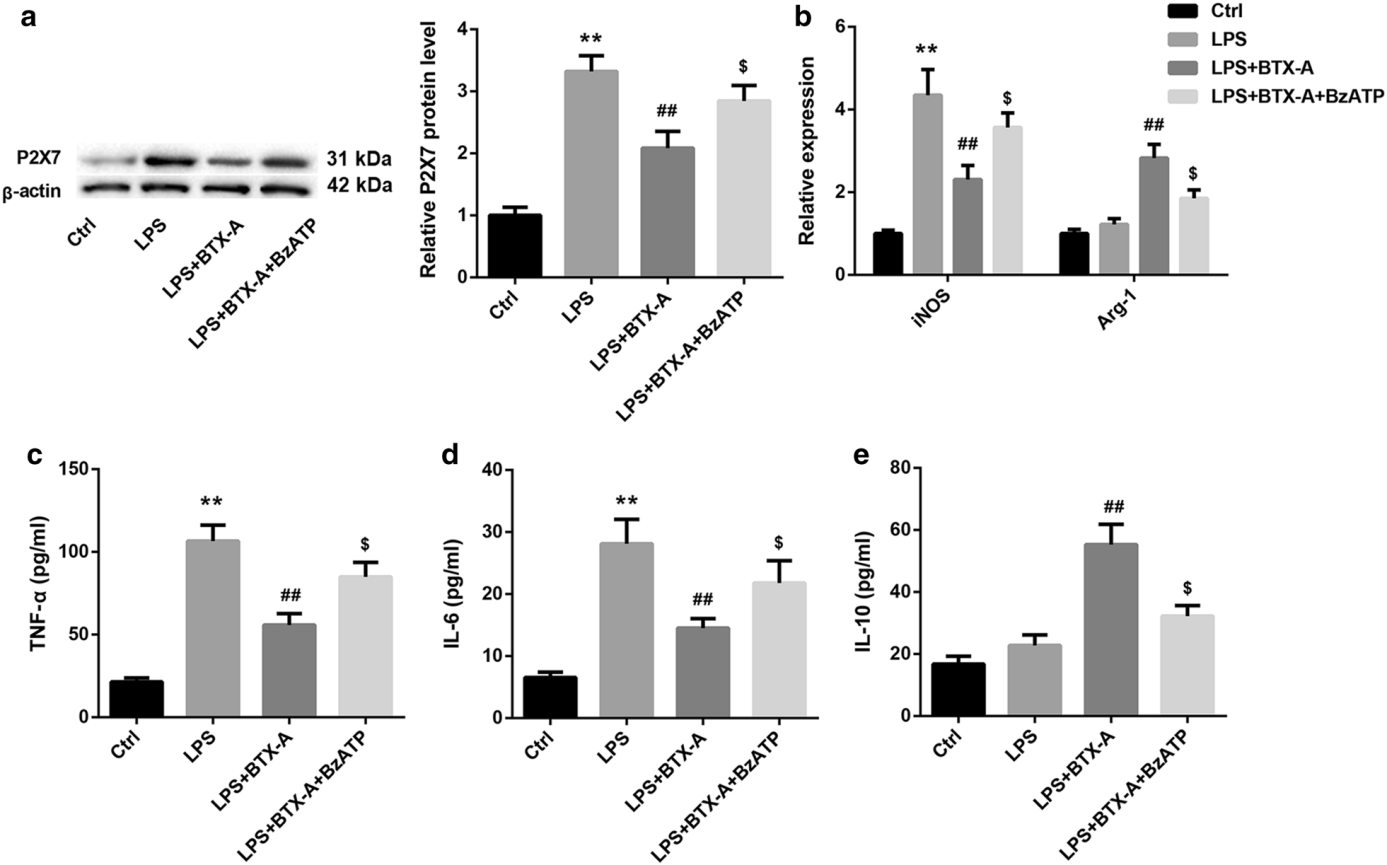
P2X7 agonist BzATP attenuated the BTX-A-mediated promotion of microglial M2 polarization. **a** Western blot analysis of P2X7 protein level, **b** relative mRNA levels of iNOS and Arg-1 determined by qRT-PCR, and levels of M1-related TNF-α (**c**) and IL-6 (**d**) and M2-related IL-10 (**e**) in HAPI cells in the groups of Ctrl, LPS, LPS + BTX-A, and LPS + BTX-A + BzATP. BTX-A: 0.1 nM; LPS (100 ng/mL) was used as a microglial activator; BzATP (200 μM) was used as a P2X7 agonist. ^**^P < 0.01, vs. Ctrl; ^##^P < 0.01, vs. LPS; ^$^P < 0.05, vs. LPS + BTX-A

To elucidate whether BTX-A promotes microglial M2 polarization through decreasing P2X7 expression, we transfected HAPI cells with pcDNA3.1-P2X7 to overexpress P2X7, followed by treatment with LPS and BTX-A. P2X7 overexpression could significantly abrogate the BTX-A-mediated downregulation of P2X7 under LPS stimulation, leading to P2X7 upregulation (Fig. 6a). Of note, BTX-A-mediated downregulation of M1 markers (iNOS, TNF-α and IL-6), and upregulation of M2 markers (Arg-1, IL-10) under LPS stimulation were significantly attenuated by P2X7 overexpression (Fig. 6b–e). Furthermore, P2X7 agonist BzATP yielded similar effect in comparison with P2X7 overexpression (Fig. 7). These data together indicated that BTX-A promoted microglial polarization to the M2 phenotype through suppressing P2X7.

## Discussion

In the present study, we found that the MWT and TWL values in NP model rats were lower than those in the sham-operated rats, whereas BTX-A administration elevated the MWT and TWL in NP model rats. These findings confirmed the relief of NP by BTX-A. The analgesic effect of BTX-A in NP in the current study was supported by previous studies [16, 17].

Activated microglia in the spinal cord in response to nerve injury can enhance the synaptic transmission of spinal dorsal horn neurons through various cell surface receptors and pro-inflammatory factors, and thus participate in the occurrence and development of NP [6]. In the present study, we observed a significant increase in Iba-1^+^ cells (microglial marker) in rat L4–L6 spinal cord segments following CCI, indicating microglial activation following NP. Our results were consistent with previous studies [23, 24].

Then we explored whether the analgesic effect of BTX-A was associated with its regulation of microglial polarization. Evidence suggests that microglial M1 polarization and the subsequent neuroinflammation play a vital role in the development of NP. Piotrowska et al. [11] reported that chronic intrathecal administration of maraviroc diminished NP symptoms and nociceptive threshold as well as decreased levels of pro-inflammatory cytokines in Wistar rats post-CCI. Convincing researches have indicated that switching microglial polarization from the pro-inflammatory M1 phenotype to the anti-inflammatory M2 phenotype represents a novel therapeutic strategy for NP [10–13]. For example, Willemen et al. [13] proposed that persistent hyperalgesia in GRK2 (G protein-coupled receptor kinase)-deficient mice was associated with an increased ratio of M1/M2 type markers in spinal cord microglia/macrophages. Intrathecal miR-124 treatment could alleviate NP by restoring spinal M1/M2 markers, indicating that the phenotypic transition of M1/M2 microglia is critical for the relief of NP. To our knowledge, this study is the first to demonstrate that BTX-A administration induces microglial polarization toward the M2 phenotype.

Further in vitro assays in this investigation confirmed that BTX-A treatment promoted microglial M2 polarization in LPS-stimulated rat HAPI microglial cells. P2X7 receptor is highly expressed in the DRG of NP rats and is implicated in the pain transmission and the occurrence of NP [19, 25]. Silencing of long non-coding RNA NONRATT021972 has been shown to reduce mechanical and thermal hyperalgesia in diabetic rats through decreasing the P2X7 receptor in DRG [19]. It was reported that P2X7 receptor plays a role in regulating microglial M1/M2 polarization. Inhibition of P2X7 receptor could inhibit microglial M1 polarization in ischaemic conditions [22]. To our knowledge, the current study provided the first evidence that BTX-A promotes microglial M2 polarization by decreasing P2X7 expression in LPS-stimulated HAPI cells.

## Conclusions

In conclusion, this study demonstrates that BTX-A promotes microglial M2 polarization and suppresses CCI-induced NP through suppressing the P2X7 receptor. These findings provide new insights into the mechanism of BTX-A in relieving NP.

## Supplementary information

**Additional file 1: Fig. S1.** Immunofluorescence double-labeling of P2X7 and CD11b or MAP2 in rat L4-L6 spinal cord segments. MAP2 for neuron, CD11b for microglia; DAPI for nuclear. Scale bar, 25 μm.

## Data Availability

The datasets used and/or analysed during the current study are available from the corresponding author on reasonable request.

## Abbreviations

Arg-1: Arginase-1
BTX-A: Botulinum toxin type A
CCI: Chronic compression injury
CCK-8: Cell Counting Kit‐8
CD206: Mannose receptor
DRG: Dorsal root ganglion
ELISA: Enzyme-linked immunosorbent assay
GRK2: G protein-coupled receptor kinase
Iba-1: Ionized calcium binding adaptor molecule-1
IL: Interleukin
iNOS: Inducible NO synthase
MAP2: Microtubule-associated protein 2
MWT: Mechanical withdrawal threshold
NP: Neuropathic pain
P2X: Purinergic ion channel receptor
qRT-PCR: Quantitative real-time PCR
SD: Standard deviation
SPF: Specific pathogen-free
TNF: Tumor necrosis factor
TWL: Thermal withdrawal Latency
