# Supplementary figures and images for: Botulinum toxin type A promotes microglial M2 polarization and suppresses chronic constriction injury-induced neuropathic pain through the P2X7 receptor
Source: Cell Biosci. 2020 Mar 23;10:45. doi: 10.1186/s13578-020-00405-3 (PMC7092425; doi:10.1186/s13578-020-00405-3)

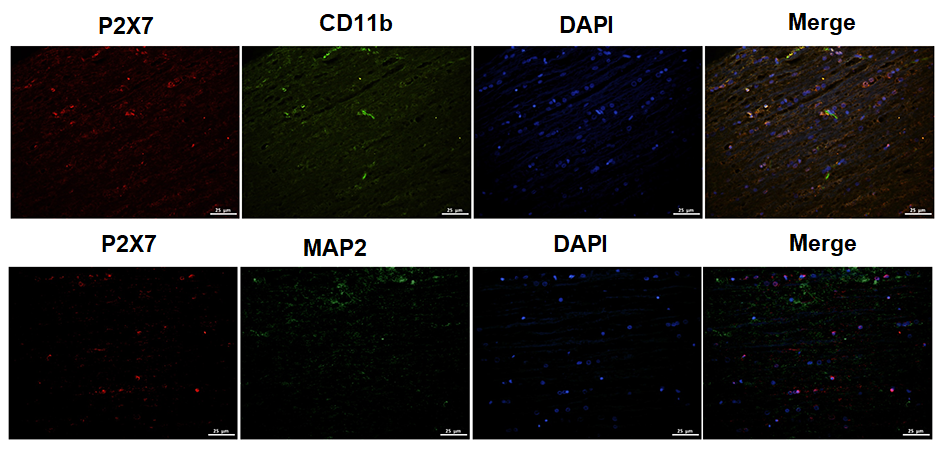

Supplement: Supplementary file 1 — Additional file 1: Fig. S1. Immunofluorescence double-labeling of P2X7 and CD11b or MAP2 in rat L4-L6 spinal cord segments. MAP2 for neuron, CD11b for microglia; DAPI for nuclear. Scale bar, 25 μm. [file 13578_2020_405_MOESM1_ESM.tif]
